# Data sharing in clinical trials – practical guidance on anonymising trial datasets

**DOI:** 10.1186/s13063-017-2382-9

**Published:** 2018-01-10

**Authors:** Catriona Keerie, Christopher Tuck, Garry Milne, Sandra Eldridge, Neil Wright, Steff C. Lewis

**Affiliations:** 1 Edinburgh Clinical Trials Unit, Usher Institute of Population Health Sciences and Informatics, University of Edinburgh, Nine Bioquarter, 9 Little France Road, Edinburgh, EH16 4UX UK; 2 Queen Mary University of London, London, UK; 3 CTSU - Clinical Trial Service Unit and Epidemiological Studies Unit University of Oxford, Oxford, UK

**Keywords:** Data sharing, Anonymisation, Clinical trial, Controlled access, Direct identifier

## Abstract

**Background:**

There is an increasing demand by non-commercial funders that trialists should provide access to trial data once the primary analysis is completed. This has to take into account concerns about identifying individual trial participants, and the legal and regulatory requirements.

**Methods:**

Using the good practice guideline laid out by the work funded by the Medical Research Council Hubs for Trials Methodology Research (MRC HTMR), we anonymised a dataset from a recently completed trial. Using this example, we present practical guidance on how to anonymise a dataset, and describe rules that could be used on other trial datasets. We describe how these might differ if the trial was to be made freely available to all, or if the data could only be accessed with specific permission and data usage agreements in place.

**Results:**

Following the good practice guidelines, we successfully created a controlled access model for trial data sharing. The data were assessed on a case-by-case basis classifying variables as direct, indirect and superfluous identifiers with differing methods of anonymisation assigned depending on the type of identifier. A final dataset was created and checks of the anonymised dataset were applied. Lastly, a procedure for release of the data was implemented to complete the process.

**Conclusions:**

We have implemented a practical solution to the data anonymisation process resulting in a bespoke anonymised dataset for a recently completed trial. We have gained useful learnings in terms of efficiency of the process going forward, the need to balance anonymity with data utilisation and future work that should be undertaken.

**Electronic supplementary material:**

The online version of this article (doi:10.1186/s13063-017-2382-9) contains supplementary material, which is available to authorized users.

## Background

There are good reasons why researchers should share their data with others. Existing research data can be used to answer questions beyond those planned in the original study, to analyse outcomes that were not included in the primary analysis, to enable individual participant data meta-analysis, and to investigate new methodologies for analysing data. In addition, sharing allows for transparency as published results can be independently validated. It is cheaper and more efficient to use existing data than to collect additional data, and puts fewer patients at risk. Funders and publishers have updated their policies to encourage or require data sharing at the participant level [1], and there is an increasing pressure to share data.

Other pressures on researchers discourage them from sharing data. For example, results counter to the sponsor/funder expectations or the possibility of future publications may result in a researcher wishing to keep the data private. Data ownership could also be considered a grey area which may prevent the data-sharing mechanism being implemented. Confidentiality of personal information is covered in the UK by the Common Law and the Data Protection Act (1998) [2] which follows the EU Data Protection Directive (1995). Data protection laws exist in countries outside the EU, with similar protections. The UK Common Law and Data Protection Act covers identifiable data on living individuals, and data that could be identified from other information that the data controller has or is likely to get. If you have specific consent to share personal data, you can share it (as specified by the consent). If the participants have died, they are not subject to the Data Protection Act. Otherwise, data can only be shared if they are anonymised and the data are not identifiable – and identifiability has to take into account identification that could be performed through information that the data controller has or is likely to get. Working through these issues takes time, which may also put researchers off sharing their data.

The pressure to share data can viewed as both positive and negative, but should be underpinned by the need to ensure patient trust at all times [3, 4]. Trying to balance these pressures leaves researchers caught in the middle. Guidance on data sharing is available [5–10] and there is an increasing interest in determining the best methodology for carrying out these processes [11, 12]. For researchers, such as ourselves, working in academic trials units in the UK, the MRC HTMR guidance [5] is particularly useful. The MRC HTMR guidance says that at the end of a trial, trialists should prepare an anonymised dataset ready for sharing, having determined an appropriate level of anonymisation. The dataset preparation should be done by individuals with an understanding of data management and basic statistics, and there should be independent quality control. The dataset should be in a form recognised by a range of software. The pack for sharing should include supporting documentation including the protocol and annotated data collection forms (including any amendments throughout the study). Although much has been written, there remain gaps in the detail of exactly what researchers need to do to share data safely. In particular, the process of anonymisation is not described in sufficient detail.

It is very hard to completely anonymise data while still leaving it in an analysable form. Relatively simple facts, such as age and country of residence, can identify a person if they are exceptionally old – the names, dates of birth and countries of the world’s oldest living people are published in Wikipedia [13]. However, it is unlikely that one of these people will be in a particular research dataset, but there are an infinite number of other rare combinations of patient characteristics; and the amount of information available publicly is ever-growing. As you remove information from a dataset, you remove informative detail that is useful in analysis.

This paper describes in detail methods for creating an anonymised dataset with reference to a recently completed trial conducted within the Edinburgh Clinical Trials Unit (ECTU).

## Methods

The MRC HTMR Good Practice Principles for sharing individual participant data [5] formed the starting point in creating an anonymised dataset. The main dataset used as an example was the TOPPIC trial [14] – a double-blind, parallel-group randomised trial, investigating whether mercaptopurine (MP) can prevent or delay post-operative recurrence of Crohn’s disease. This trial commenced in 2008 and recruited 240 patients across 29 UK hospitals over a period of 49 months with a 3-year follow-up.

All decisions and discussion points were recorded throughout the process and are described in the following ‘Results’ section.

## Results

The following is a detailed description of the process of creating an anonymised dataset.

### Decide whether to use an open-access or controlled-access model

Data can be shared using an open-access or a controlled-access model. In an open-access model, the dataset is made public, and can be downloaded by anyone, with no restrictions. In a controlled-access model, the data are only released if certain conditions are met, for instance if the requestor can prove that they are a bona fide researcher with a sensible question to answer, and if appropriate data-sharing agreements are signed. Open access is riskier, and a higher level of anonymisation is necessary to ensure that the data are not considered personal data and subject to the Data Protection Act. Unfortunately, as the level of anonymisation increases, the level of data utility decreases, and it may not be possible to use an open-access model and retain all the detail to enable someone to repeat the final analysis exactly. We favour controlled access, as recommended by the MRC HTMR guidance [5] for several reasons. By requiring application, researchers have to propose a hypothesis and, therefore, can avoid data dredging. Data access agreements with recognised institutions and bona fide researchers can form part of the anonymisation process and should reduce the risk of any malicious re-identification. Lastly, a controlled-access model will ensure that the original work is credited.

### Assemble initial data-sharing pack

The initial data-sharing pack includes the study protocol, annotated data collection forms, the statistical analysis plan (SAP), final report and data dictionary, noting any relevant amendments to these during the study. The annotated data collection forms show the variable name and data table for every data point collected. This information is also available in tabular form in the data dictionary, but the data dictionary also details the data type of each variable (e.g. numeric, text). The data dictionary is amended through the anonymisation process. Although statistical analysis programmes can be included in the data-sharing pack, we have not added them, as they were not prepared assuming that they would be publicly accessible. However, it should be noted, that there is value in including statistical analysis programmes in a data-sharing pack. Their inclusion can benefit secondary researchers in terms of learning and can also reduce analysis time following a data-sharing request.

### Identify direct identifiers, indirect identifiers and superfluous information

The first stage of the data anonymisation process is to consider every variable and assess whether it is a direct identifier, an indirect identifier, or superfluous. Hrynaszkiewicz [15] lists 28 types of potential participant identifiers, split into direct and indirect identifiers (Table 1). The direct identifiers are very likely to either immediately identify a participant or pose a very real risk that individuals can be identified. Indirect identifiers may pose a risk in combination with others listed. Problem variables must either be removed or modified, so that the risk of identification is diminished. Superfluous data, such as audit trail data, should be removed.

**Table 1:** Aggregated list of potential patient identifiers in datasets (Hrynaskiewicz [15])

Direct identifiers	Indirect identifiers
01. Name	A. Place of treatment or health professional responsible for care
02. Initials	B. Sex
03. Address, including full or partial postal code	C. Rare disease or treatment
04. Telephone or fax numbers or contact information	D. Sensitive data, such as illicit drug use or ‘risky behaviour’
05. Electronic mail addresses	E. Place of birth
06. Unique identifying numbers	F. Socioeconomic data, such as occupation or place of work, income, or education
07. Vehicle identifiers	G. Household and family composition
08. Medical device identifiers	H. Anthropometry measures
09. Web or internet protocol addresses	I. Multiple pregnancies
10. Biometric data	J. Ethnicity
11. Facial photograph or comparable image	K. Small denominators – population size of < 100
12. Audiotapes	L. Very small numerators – event counts of < 3
13. Names of relatives	M. Year of birth or age
14. Dates related to an individual (including date of birth)	N. Verbatim responses or transcripts
Superfluous	
02. Superfluous information (audit trail data, administration data)	

The original data dictionary is used as the starting point for documentation of the anonymisation process with each variable assigned a value. Direct identifiers are given the values 01–14, indirect identifiers are given the values A–N and superfluous information is given the value 15 (Table 1).

### Assign methods of anonymisation for direct identifiers

Variables coded as direct identifiers were assigned to two categories:

1. Delete
  For the TOPPIC trial, all names, initials, addresses (including email) relating to patients, relatives and study personnel were removed from the anonymised database
2. Modify

#### Unique identifiers

A unique identifier for each trial participant will always exist and very often the original unique identifier assigned can be linked to study sites. Therefore, all unique identifying numbers (e.g. subject number, pre-screening identifier) were recoded using random number generator methodology which ensured reproducibility and linkage to the original unique identifier (see Additional file 1). The MRC HTMR guidance suggests that the link between the new code number and the original unique identifier should be destroyed. For the TOPPIC trial, the link has been maintained in case of any queries relating to the anonymised dataset from secondary researchers.

#### Other identifier values

Identifier numbers relating to laboratory samples for individual patients were recoded similarly, while ensuring that the sample identifier could be linked back to the correct participant. This also applied to adverse event identifiers which were required to be linked to the patient and any associated drug schedule changes resulting from an adverse event. Bottle codes forming part of the prescription process were also recoded in this manner.

#### Dates

All dates relating to individuals (including date of birth) were classed as direct identifiers. For anonymisation purposes, date of randomisation was used as a reference date for each participant, classed as day 0.

Complete dates (i.e. those where a day, month and year are provided) were modified to be relative to day 0. For example, a date of randomisation of 15 January 2014 with a date of admission to the trial of 16 January 2014 gives a new study day admission to trial of 1.

Partial dates are very often captured, particularly in relation to start and stop dates for concomitant medications or adverse events. Most commonly the month and/or year are captured, but not the day, e.g. May 2017, or just 2009. There are a few solutions to this – the date could be removed completely, only partial dates could be removed or a reduced version of the days relative to randomisation date method could be employed.

For the TOPPIC trial, it was decided that only partial dates would be removed, i.e. if the day and/or month part were unknown, days relative to randomisation was not captured. So, if there were 25 patients with missing day of medication start, then these 25 dates were removed. There was an exception to this rule for the primary and secondary time-to-event outcomes. Capturing an accurate date is critical for a time-to-event outcome. There were two instances where secondary endpoint dates were captured as month and year only. For these occurrences, it was decided that the missing day would be imputed as 15 (i.e. mid-month) in order that the two patients with a partial secondary endpoint date would not be excluded from the secondary outcome analyses. This was in line with how partial dates were handled in the original statistical analysis of primary and secondary outcomes.

### Assign methods of anonymisation for indirect identifiers

The second pass of the data dictionary involves the indirect identifiers, those that may present a risk if present in combination with others. To decide if these needed anonymisation a consensus model was used comprising a trial manager, a statistician and an IT programmer. Some of the fields (especially those that could potentially have small event counts), were summarised to help assess the risk.

Variables coded as direct identifiers were assigned to three categories:

1. Delete
  Indirect identifiers which could be considered a rare disease or treatment (category C) or have a low event count (category L) were assessed on an individual basis. Where the disease or treatment occurred in only one patient, it was decided that this field should be removed completely. Where the frequency was greater than two, clinician input was sought to determine whether the rarity of the disease or treatment could lead to identification of individuals.
  Verbatim responses or transcripts were removed since free text can very often reveal personal patient information. All comments were removed, as were any descriptions (e.g. adverse event descriptions, reasons for stopping medication, family history, physical examination descriptions) and fields relating to additional information.
  Any location-related fields other than study site/centre were removed, e.g. names of hospitals, location of treatments, place of birth.
2. Modify
  Study sites/centres were modified in line with the methods employed for subject number using a random-number generator approach.
  Year of birth or age could be considered to be indirect identifiers. For TOPPIC, age was categorised into a small number of groups (i.e. below 16 years, between 17 and 40 years, above 40 years). This field was retained in the database as it posed no risk of patient identification.
3. Leave
  Gender was classed as an indirect identifier, but was retained in the database. This should be assessed on a trial-by-trial basis as a disproportionate number of men or women within a trial may lead to patient identification.
  Continuous and ordinal study outcomes were checked for outliers before making the decision that identification of patients would not be possible and that these outcomes could remain in the data dictionary. These included patient scores (e.g. quality of life), clinical measurements (e.g. vital signs), laboratory samples and baseline characteristics (e.g. height, weight).

### Other issues

Sometimes it is necessary to separate out an entire table of sensitive data with a view to these data being shared only where a specific request has been made and justification given. For TOPPIC, the pregnancy data fell into this category and no pregnancy-related details were shared in the anonymised database.

For TOPPIC, a serious adverse event (SAE) was recorded which related to the child of a patient born during the TOPPIC follow-up period. Several feasible solutions were presented: (1) anonymise the SAE record, but link it to the TOPPIC patient (parent), (2) anonymise the SAE record and destroy the link to the TOPPIC patient and (3) remove the data completely. It was decided that this SAE record should be anonymised, but the link still retained to the patient with a flag to denote that this SAE related to the child (solution 1). This way, individuals would remain unidentified, but this rare event would be retained within the anonymised data.

Any data/tables relating to trial management were removed from the anonymised database as these were utilised purely for day-to-day running of the trial. These included monitoring schedules, missing data and tracking logs (e.g. blood tests taken), duty rotas, email records, non-recruited patient logs and recruitment targets.

#### Experience from other anonymised datasets

Other trials going through the same process within ECTU are BIDS [16] and GaPP [17], both of which have been published and have provided valuable learnings in the data anonymisation process, particularly demonstrating that a standardised solution may not always be the most appropriate.

The BIDS database captured several time-based fields (i.e. hours and/or minutes) which could be potentially identifiable. This aspect of the data does not quite fit with the list of potential identifiers, as the list only referred to dates related to an individual, not times. The potential to create study minutes in the same way as for study days was discussed, but based on the date and time of randomisation. A disadvantage to this is that some data utility could be lost as secondary researchers would not then know the time of day. In this instance, it was felt that creating study minutes would not particularly add in terms of anonymisation as dates are already being modified to a study day. A decision was made to not modify the hours and minutes component. If this was an open-access model study minutes may have been created to make the data less identifiable.

For the GaPP anonymisation process, the decision was taken to merge some categorical fields in order to prevent patient identification. Adverse events classed as mild or moderate were combined into one category. Similarly marital status categories of separated or divorced were combined into a single group.

Other anonymisation complexities to consider are trials researching genetic conditions where a ‘family identifier (ID)’ link within an anonymised environment must be maintained. The ZiPP [18] trial currently being undertaken through ECTU is one such study. This family ID is an important aspect of the statistical analyses in terms of the requirement to account for clustering (within family) effects and is an area we need to give consideration to for anonymising this data in the future.

### Create the final dataset and data dictionary

On completion of the data anonymisation process, the dataset was exported to delimited text format (i.e. comma-separated variables (csv)). This can easily be read into widely available packages such as Microsoft Excel. It has the advantage that it will continue to be readable long into the future, whereas other data tables are not readable as software versions are updated over time. However, an exception to this would be where values contain commas (e.g. if there are any text fields).

The folder where these data are stored should be protected so that they cannot unintentionally be altered. Data dictionary production can be automated, but for the TOPPIC trial this was a retrospective and relatively manual process.

### Check the anonymised dataset

1. Is it accurate?
  As a check to the anonymised dataset, the ‘gold standard’ is to independently re-run the analyses with the modified dataset. This was done for the TOPPIC trial by the original trial statistician, concentrating on the primary and secondary outcomes only. Minor modifications to the original analysis programmes were required in order that they would run correctly with the anonymised data. Corresponding analyses outputs were created in PDF format and checked against the outputs from the original statistical report as evidence that the anonymisation process had created a dataset which could replicate the analyses.
  The primary and secondary outcomes were both time-to-event in nature. As noted earlier, capturing an accurate date is critical for a time-to-event outcome and for TOPPIC there were two instances where secondary endpoint dates were captured as month and year only. Initially, these partial dates were set to missing in the anonymisation process rather than the day being imputed as 15, as had been the case for the original statistical analyses. This caused problems with the replication of results for the secondary outcome as these two records were excluded from the anonymised analysis re-run, but had been included in the original analyses. Discussion between the statistician and database programmer rectified this minor issue and the partial dates were imputed in the sharable dataset as mid-month (i.e. 15) in order that the original statistical analyses could be replicated.
  A cheaper alternative to a complete re-run of the original analyses would be to check a few key facts from the main analysis, and use simple automated checks for the rest. In the programming of the anonymised version of the dataset, it is more likely that an incorrect variable will be used in place of the intended one, rather than an individual point in the dataset being altered. Thus, comparing the difference between the maximum and minimum values in the anonymised and original datasets is likely to be sufficient for continuous variables and dates.
2. Is it anonymous?
  A motivated intruder test, as suggested by the Information Commissioners Office [7], can be used to determine whether the modifications performed renders the data anonymous in such a way that individual participants are no longer identifiable. In its simplest form, a person who starts without any prior knowledge is provided with the dataset and would attempt to identify individuals from the dataset. It is assumed that the motivated intruder is reasonably competent and has access to public information, but has no specialist skills. This work is time-consuming and probably practically beyond the scope of most research groups given their workloads and budgets.

### Release

On completion of the process, a method for release was devised. The data-sharing pack was created which included the anonymised data, the data dictionary, the SAP, data collection forms and the final protocol. All items were considered to be freely available with the exception of the anonymised dataset.

This data-sharing pack was then uploaded to the University of Edinburgh data repository, DataShare [19] which is based on an open digital repository, DSpace [20]. This in turn creates a Digital Object Identifier (DOI) which can be formally referenced.

Once the dataset is uploaded to DataShare, a secondary researcher can apply to access the data through the DataShare system. This sends an email request to a shared inbox. Once a request is received a simple request form is sent to the secondary researcher to complete (see Additional file 2). The application is reviewed by a committee and, if approved, a data-sharing agreement is signed between the institutions of the data owner and the secondary researcher. To release the data, the request is approved on DataShare and the anonymised dataset is emailed to the requester.

To aid transparency, all request forms and the decisions made by the committee are published on the ECTU website.

## Discussion

### Strengths

Based on MRC guidance, a practical solution has been presented to create an anonymised dataset which could be applied across all clinical trials units (CTUs). We have demonstrated, by means of replication of the TOPPIC primary and secondary outcome analyses using an anonymised dataset, that anonymisation can be balanced with data utility and practicality. Generic rules can be created for direct identifiers and decisions on indirect identifiers can be made on a trial-by-trial basis. The resulting solution is relatively straightforward and could easily be implemented either by a statistician or a database programmer.

Through this process, we have been able to create a generic data-sharing agreement that can be tailored for individual studies. The data-sharing process has also been agreed in principle with the TOPPIC trial sponsor.

### Limitations

Since this was the first time that we had undertaken a data-sharing process, it took a relatively long time. Each dataset (and, in many cases, each data variable within the dataset) was assessed individually by the team through joint discussion. With experience, the process will get quicker, but the more complex the anonymisation process, the longer it takes and there is a likelihood of errors arising. As with any bespoke process, there is a time and cost impact, particularly with regard to the controlled-access model chosen here. By contrast, an open-access model is much cheaper. Furthermore, the controlled-access model can be problematic with respect to ongoing maintenance if, for instance, no one is retained at the institution that worked on the original study, email addresses cease to exist, etc. Long-term feasibility should be taken into consideration when designing the access model that will be used within an organisation. Indeed, we have a generic trials unit data-sharing email address that does not depend on a single individual from a trial continuing to be employed. Our processes are being designed to work long after the original trial team ceases to exist.

One final limitation is that the exercise could also be viewed as moderately subjective, but by working collaboratively across disciplines, we have worked around this by achieving a consensus on all decisions taken.

### When should this work be done?

The MRC guidance states that dataset preparation can either be proactive (prepared in advance) or reactive (prepared when a request is made). We suggest that a proactive model is preferred. The process will be quicker if all this is done at study start-up, rather than at the end. In addition, it takes a long time to prepare and check a dataset, and data requestors are likely to be frustrated if they have to wait months to receive the data.

As for the specific process of data anonymisation, the exercise could be split into two separate parts – a first stage of anonymising direct identifiers, followed by a second stage of determining indirect identifiers at a separate point in time. If preferred, all anonymisation can be performed at the end of the second stage. This was the case for the TOPPIC trial.

### Future work

Future work should concentrate on refining the model to reduce resource implications in terms of time and cost. As all CTUs develop their own data-sharing models, there will be increased interest in creating an efficient process that is relatively straightforward to follow. Work is ongoing to demonstrate the time and cost involved in such an exercise [21].

Going forward, the process should be improved and refined. Consideration should be given to appropriate wording for future consent and patient information leaflets within a trials environment and steps are being taken to address this in line with current recommendations [3]. There is also a requirement to check copyright on the data collection forms, particularly questionnaires, prior to release.

There may be a need to create a formal data-sharing committee together with a concerted effort to advertise availability of data. Appropriate documentation should be embedded in formal processes for all CTUs (e.g. via Standard Operating Procedures (SOPs)). This future work will also have resource implications, but should result in a positive step forward in the data-sharing domain.

## Conclusions

We think that the MRC guidance can provide a solution for CTUs that want or need to share data. We will be able to create generic rules for direct identifiers, but indirect identifiers will probably need a per-trial approach. As mentioned before, balancing the requirement of anonymisation with the need for data utility is not always easy to achieve. The practicality of the anonymisation process should remain a high priority. Going forward, we will become more adept at recognising the types of data that regularly occur within our trials and how these should be best handled for data sharing, perhaps via an automated process. However, there will always be unusual cases within each trial which may mean full automation is not possible.

Data sharing is a balance of mitigating risk against encouraging good use. No system is perfect but it is imperative that we develop this aspect of trials reporting in order to progress towards a fully compliant and robust end-to-end process.

## Additional files

Additional file 1: Methods employed for recoding of unique patient identifiers. (DOCX 12 kb)

Additional file 2: Data Request Form v1.0 06Jan2017. (DOCX 18 kb)

## Abbreviations

AUKCAR: Asthma UK Centre for Applied Research
BIDS: Trial acronym for ‘Bronchiolitis of Infancy Discharge Study’
csv: Comma-separated variable
CTU: Clinical trials unit
DOI: Digital Object Identifier
ECTU: Edinburgh Clinical Trials Unit
GaPP: Trial acronym for ‘A Pilot Randomised Controlled Trial of the Efficacy and Mechanism of Action of Gabapentin for the Management of Chronic Pelvic Pain in Women’
MRC HTMR: Medical Research Council Hubs for Trials Methodology Research
SAE: Serious adverse event
SAP: Statistical analysis plan
SOP: Standard Operating Procedure
TOPPIC: Trial acronym for ‘Randomised Controlled Trial of 6-Mercaptopurine Versus Placebo to Prevent Recurrence of Crohn’s Disease Following Surgical Resection’
UKCRC: United Kingdom Clinical Research Collaboration
UoE: University of Edinburgh.
