# Supplementary material for: Data sharing in clinical trials – practical guidance on anonymising trial datasets
Source: Trials. 2018 Jan 10;19:25. doi: 10.1186/s13063-017-2382-9 (PMC5763739; doi:10.1186/s13063-017-2382-9)
Supplement: Supplementary file 1 — Methods employed for recoding of unique patient identifiers. (DOCX 12 kb) [file 13063_2017_2382_MOESM1_ESM.docx]

**Additional File 1 – Methods employed for recoding of unique patient identifiers**

**Description**

A new table was created *tbla_KeyData.* This table held the *SubjectNo, a_subjectno* (re-coded random number) *and WhenRandomised* fields. This was then used when linking the existing tables to produce the anonymised tables containing the *a_SubjectNo* value in place of the *SubjectNo,* and using the *WhenRandomised* field to calculate the replacement date fields*.*

**Code**

SQL code for random number generation shown below:

SELECT

subjectNo,

(RAND(CAST( NEWID() AS varbinary ))) AS a_SubjectNo

FROM

tblSubjectRandomisationData p

ORDER BY subjectNo ASC
