# Supplementary material for: Data sharing in clinical trials – practical guidance on anonymising trial datasets
Source: Trials. 2018 Jan 10;19:25. doi: 10.1186/s13063-017-2382-9 (PMC5763739; doi:10.1186/s13063-017-2382-9)
Supplement: Supplementary file 2 — Data Request Form v1.0 06Jan2017. (DOCX 18 kb) [file 13063_2017_2382_MOESM2_ESM.docx]

| **Details of trial being requested** |  |
| --- | --- |
| Name of trial |  |
|  |  |
| **Data Requester** |  |
| Name of applicant |  |
| Position of applicant |  |
| Date of request |  |
| Institute where research will be conducted |  |

| **Description of the research project** |  |
| --- | --- |
| Title | |
|  | |
| Background/rationale *(maximum 200 words)* | |
|  | |
| Objectives *(maximum 200 words)* | |
|  | |
| Endpoints, analysis plan and statistical methods *(maximum 300 words)* | |
|  | |

| **Practical details of the project** |  |
| --- | --- |
| Expected start date (dd/mm/yyyy) |  |
| Expected completion date (dd/mm/yyyy) |  |
| Funding received or sought for the project? If yes, please provide details |  |
| Has this application been previously submitted and rejected? If yes, please provide details |  |

| **Project Outputs** |  |
| --- | --- |
| Expected number of planned publications |  |
| Expected title(s) of planned publications |  |

Please include with this application summary CVs for the data requester, scientific leader and methodologist/statistician.

By submitting this application to [ECTUdatashare@ed.ac.uk](mailto:ECTUdatashare@ed.ac.uk) you acknowledge that details of this application (including your name) will be published on the Edinburgh Clinical Trials Unit website.

*Please provide details of any person that will have access to the trial data*

| **Scientific Leader** | |
| --- | --- |
| Name |  |
| Position |  |
| Institute |  |

| **Methodologist/Statistician** | |
| --- | --- |
| Name |  |
| Position |  |
| Institute |  |

| **Additional researcher 1** | |
| --- | --- |
| Name |  |
| Position |  |
| Institute |  |

| **Additional researcher 2** | |
| --- | --- |
| Name |  |
| Position |  |
| Institute |  |
